# High-Gd-Payload P22 protein cage nanoparticles for imaging vascular inflammation

**DOI:** 10.1186/1532-429X-15-S1-O66

**Published:** 2013-01-30

**Authors:** Hisanori Kosuge, Masaki Uchida, Janice Lucon, Shefah Qazi, Trevor Douglas, Michael V McConnell

**Affiliations:** 1 Stanford University, Stanford, CA, USA; 2 Montana State University, Bozeman, MT, USA

## Background

The bacteriophage P22 protein cage can be bioengineered to contain a high-relaxivity gadolinium (Gd) payload internally and targeting ligands externally. It also enables phage-library-based identification of novel targets. Thus, P22 may have advantages for molecular/cellular imaging by MRI.

## Methods

**Figure 1:**
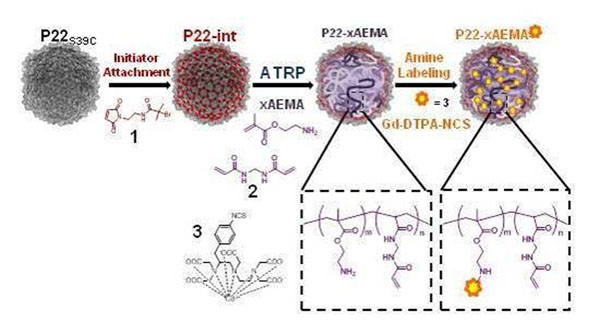


1) P22: The P22 protein cage (60 nm) is bioengineered with an internal polymer network with amine functional groups allowing incorporation of ~9100 Gd-DTPA molecules per cage via the amine groups (Figure 1: [1]). This provides a per cage relaxivity of 70000 mM^-1^s^-1^, superior to Gd-DTPA for the equivalent Gd concentration.

2) Atherosclerosis Models: Both ApoE-deficient (ApoE^-/-^) and FVB mice were used. ApoE^-/-^ mice develop atherosclerosis enhanced by high-fat diet. FVB mice develop macrophage-rich carotid lesions with carotid ligation in combination with high-fat diet and diabetes induction [2].

3) P22-polymer-Gd *in vivo* MR imaging: Mice were injected intravenously with P22-polymer-Gd (N=5, 20 µmol Gd/kg, one-fifth the typical clinical dose) or Magnevist (N=1, 20 µmol Gd/kg). Vascular MRA at 1T was performed (Aspect M2^TM^, 500 mT/m, 2500 T/m/s) using 3D-SPGR (TR/TE=12 ms/2.1 ms, slice thickness=1 mm, FOV=5 cm, matrix=128x128, FA=45). Vessel wall MRI at 3T was performed (Signa HDx, GE Healthcare, 50mT/m, 150 T/m/s) with a phased-array mouse coil (RAPID MR International), using a double inversion recovery fast spin echo sequence (TR/TE= 400 ms/15 ms, slice thickness=1mm, FOV=3 cm, matrix= 256x256) up to 24 hours after injection.

4) RGD-targeted P22 *ex vivo* fluorescence imaging: Molecular targeting of P22 was evaluated by attaching RGD peptides externally, which targets the αVβ3 integrin, upregulated on activated macrophages. ApoE^-/-^ mice (N=4) were injected intravenously with RGD^+^P22 or RGD^-^P22 (labeled with Cy5.5, 4 nmol/mouse). Forty-eight hours later, *ex vivo* fluorescence imaging was performed using Maestro^TM^ imaging system (Cri, Woburn, MA). Maximum plaque signal intensities were measured and compared.

## Results

**Figure 2:**
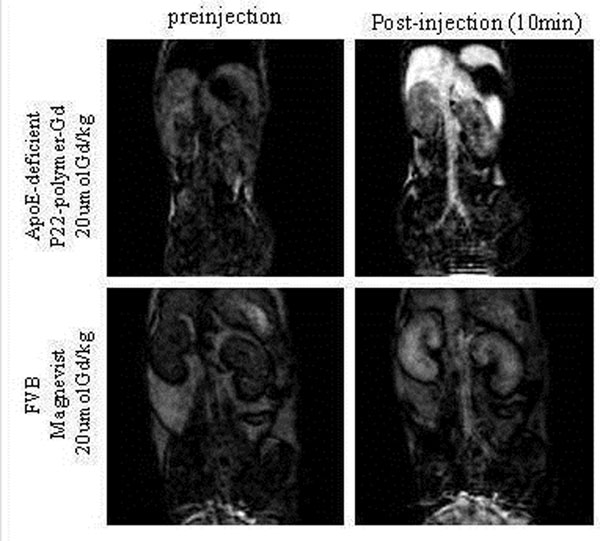


**Figure 3:**
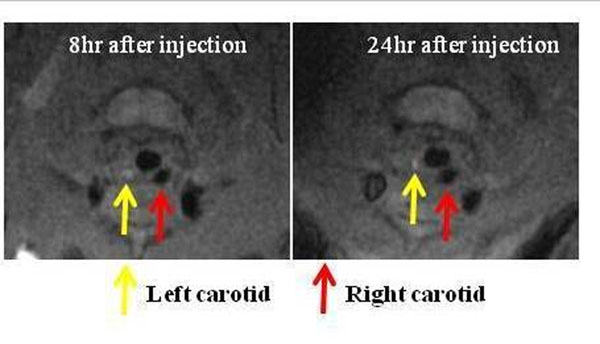


**Figure 4:**
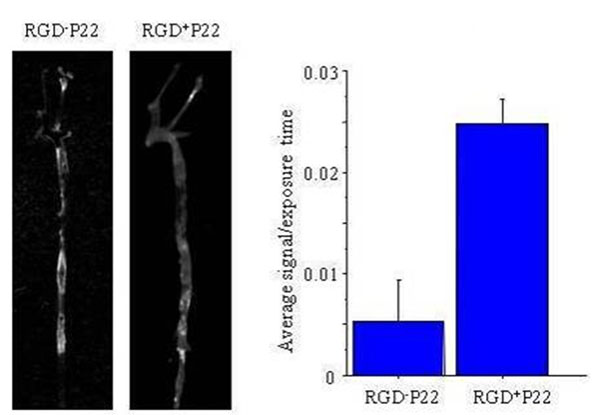


Low dose P22-polymer-Gd showed strong enhancement for 1T vascular MRA (Figure 2). It also showed clear enhancement of the aortic wall (ApoE^-/-^) and ligated carotid (FVB) at 3T (Figure 3). *Ex vivo* fluorescence imaging showed the accumulation of both RGD^+^P22 or RGD^-^P22 in atherosclerotic lesions (Figure 4). RDG targeting enhanced plaque uptake (RGD^+^P22: 0.025 ± 0.002 counts/sec vs. RGD^-^P22: 0.005 ± 0.004 counts/sec, p=0.05).

## Conclusions

The P22 protein cage nanoparticle demonstrates both internal high-relaxivity Gd-loading for *in vivo* MRI as well as external RGD-targeting for enhanced uptake in vascular inflammation. Thus, P22 is a novel, multi-functional nanoparticle platform for targeted-imaging of atherosclerosis.

## Funding

GE healthcare, Kowa, Inc.

## References

[B1] Lucon J Nat Chem 2012 4 781 788 10.1038/nchem.1442 23000990 PMC3763733

[B2] Kosuge PLoS One 2011 6 e14523 10.1371/journal.pone.0014523 21264237 PMC3021517

